# Colorectal cancer adjuvant chemotherapy trends among a nonelderly veteran cohort at a southern veterans health administration

**DOI:** 10.1002/cnr2.1508

**Published:** 2021-08-12

**Authors:** Richard L. Martin, Gretchen C. Edwards, Lauren R. Samuels, Cathy Eng, Christianne L. Roumie

**Affiliations:** ^1^ Veterans Health Administration‐Tennessee Valley Healthcare System Geriatric Research Education Clinical Center (GRECC), HSR&D Center Nashville Tennessee USA; ^2^ Department of Medicine Vanderbilt University Medical Center Nashville Tennessee USA; ^3^ Department of Surgery Vanderbilt University Medical Center Nashville Tennessee USA; ^4^ Department of Biostatistics Vanderbilt University School of Medicine Nashville Tennessee USA

**Keywords:** cancer care, chemotherapy, clinical guidelines, colorectal cancer, pathway analysis

## Abstract

**Background:**

For patients with high‐risk stage II or stage III colorectal cancer (CRC), adjuvant chemotherapy (AC) improves survival, yet use varies substantially across medical oncology settings.

**Aim:**

Utilization of guideline concordant CRC AC was assessed at a Veterans Health Administration (VHA) facility to determine quality improvement initiatives.

**Methods and Results:**

The study was a retrospective review of CRC surgeries from January 1, 2000 to December 31, 2015 at a South Regional VHA. Inclusion criteria consisted of pathologic high‐risk stage II or stage III CRC, with exclusion for age ≥80, age ≥75 hospitalized with major co‐morbidity in the prior year, and death or discharge to hospice within 30 days of the index surgery. The primary predictor was year‐group; partitioned 2000–2005, 2006–2010, 2011–2015 to account for changes in NCCN high risk stage II definitions. Primary outcome was AC receipt. Secondary outcome was reason for chemotherapy omission. Among 180 eligible surgeries (121 colon and 59 rectal cancers), patients were mostly male (96%), white (79%) and with median age 64 years. Overall, 117 (65%) received AC. Compared to 2000–2005, patients undergoing surgery between 2011 and 2015 were less likely to receive AC (odds ratio 0.35; 95% confidence interval [CI] 0.14–0.82), due to more patients declining AC (27% vs. 6%, *p* < .01) in the NCCN eligible cohort (*N* = 180), and (32% vs. 8%, *p* < .01) in an analysis of patients who completed appointments and had AC recommended by providers (*N* = 146).

**Conclusions:**

Survival benefitting AC decreased over time among a nonelderly Veteran cohort eligible for AC. Evaluating care decisions and trends within other VHA facilities and outside the VHA are warranted.

## 1 INTRODUCTION

Approximately half of the 145 600 patients diagnosed with colorectal cancer (CRC) each year will present with high‐risk stage II or stage III disease.^1^ For these patients, 5‐fluorouracil (5FU) based adjuvant chemotherapy (AC) improves disease‐free survival (DFS) and overall survival (OS).^2^, ^3^, ^4^ Compared to observation alone, 5FU/leucovorin (LV) demonstrated a 12% absolute risk reduction [ARR] in overall survival yielding a Number Needed to Treat [NNT] = 8.4.^5^ Adding oxaliplatin to 5FU/LV (FOLFOX) improved overall survival compared to 5FU/LV, however, the magnitude of benefit was substantially smaller; ARR 4.2% and NNT = 24 for stage III disease and no difference for stage II disease.^6^ Despite these benefits, substantial variation (39%–98%) exists across medical oncology settings in the receipt of AC for eligible CRC patients.^7^, ^8^, ^9^

Patient factors associated with lowers odds of receiving of AC include older age, increased co‐morbidities, female sex, and nonwhite race.^9^ Healthcare‐system factors include nonprivate insurance, postoperative complications, increased distance to medical oncology facilities, and medical oncology facilities separate from the surgical facility or with low CRC volumes.^10^, ^11^, ^12^ How these factors influence decisions surrounding chemotherapy workflows remains unknown but may manifest as lower rates of medical oncology referrals, physicians recommending against AC, and patients declining AC.^13^

These associations suggest that the barriers and solutions to achieving quality CRC care may be highly specific to the system of care. For example, from 2003 to 2006, 77.5% of Veterans treated at a Veterans Health Administration (VHA) facility were referred to medical oncology and received timely AC.^8^ Compared to the north, west, and central regions, however, Veterans treated within a south regional VHA were significantly less likely to be referred to medical oncology or receive chemotherapy.^14^ Reasons for this regional difference remain uncertain. Additionally, few studies have assessed VHA AC utilization patterns over time, specifically with respect to the types of chemotherapy regimens prescribed. Given the differences in routes of administration and toxicities between regimens, assessing regimen‐level data may reveal important sub trends in prescribing patterns and chemotherapy acceptance. We evaluated receipt of AC within a south regional VHA among patients with National Comprehensive Cancer Network (NCCN) guideline‐eligible high‐risk stage II and stage III CRC. The aims of this study were (1) to describe temporal trends in the receipt of NCCN‐guideline‐concordant AC,^15^, ^16^ and (2) to identify specific workflow processes associated with omissions or delays in achieving guideline‐appropriate AC.

## 2 MATERIALS AND METHODS

### 2.1 Design and data sources

The study was a retrospective secondary data review cohort of Veterans undergoing colorectal resections at a south regional VHA between January 1, 2000 and December 31, 2015, identified using two sources: (1) Veterans Information Systems and Technology Architecture (VistA) and (2) the VHA gastroenterology surveillance initiative database. The study site was a 238‐bed tertiary care Veterans Affairs hospital. In FY2018 the site had 9313 hospital admissions and cared for 103 207 unique Veterans. Most surgical oncology care is performed at the primary campus, which hosts a weekly interdisciplinary Tumor Board. Veterans receive CRC care from multiple services including primary care, oncology, surgery, gastroenterology, radiology, and pathology. From November 2015 through November 2019, the outpatient medical oncology clinic consulted on ~1600 newly diagnosed oncology patients, averaging 4400 infusion visits per year. During the study time‐period, total neoadjuvant therapy for rectal cancer had not been adopted into practice.

The VHA Tennessee Valley Healthcare System Institutional Review Board (IRB) and Research and Development Committee approved this study, with a waiver for subject consent under secondary data review.

### 2.2 Population

Patients were identified using Current Procedural Terminology (CPT) codes for colorectal surgeries: 44140–44 147, 44 150–44 153, 44 155–44 158, 44 160, 44 204–44 208, 44 210–44 212, 45 110–45 119, 45 126, 45 160–45 170, 45 395–45 397^17^ or a VistA keyword search for terms “colon resection” or “rectal resection.” From this queried report, patients with pathology reports confirming colorectal cancer were included, while those with precancerous adenomas or metastatic disease identified prior to or on the date of surgery were excluded. For colon cancers, pathologic eligibility criteria consisted of NCCN defined high‐risk stage II or stage III disease. To account for changes in NCCN guidelines, the definition of high‐risk stage II colon cancer depended on the time‐period. From 2000 to 2010, high‐risk stage II colon cancer was defined as having at least one of the following: T4 tumors, perforation, <12 lymph nodes assessed, poorly differentiated histology, or macroscopic positive margins.^18^ For resections after 2010, lymphovascular invasion (LVI) and microscopic positive margins were added to the above criteria.^16^ For rectal cancers, eligibility criteria consisted of having any of the following: T3/T4 tumor, clinical or pathologic node positivity, or neoadjuvant chemoradiation.

Due to equivocal evidence of benefit in elderly patients and older patients with significant co‐morbidities,^19^, ^20^ we excluded patient's age ≥80 or ≥75 with an active co‐morbidity, defined as at least one organ dysfunctional co‐morbidity and an inpatient hospitalization related to that co‐morbidity in the prior 365 days. Patients without an opportunity for AC consideration due to death or hospice enrollment within 30 days of index surgery were excluded.

### 2.3 Predictor variables

The primary predictor was year of the index surgery, partitioned (2000–2005, 2006–2010, 2011–2015) to account for changes in NCCN guidelines.

### 2.4 Outcome variables

The primary outcome was receipt of any chemotherapy (yes/no). Secondary outcomes included the type of AC regimen received (5FU/LV, capecitabine, FOLFOX, CAPEOX). Exploratory outcomes regarding patient and provider reasons for omission of AC were abstracted from chart‐level information contained within documented notes.

### 2.5 Data collection and measurements

Study data were collected and managed using REDCap electronic data capture tools hosted through the VHA.^21^ REDCap (Research Electronic Data Capture) is a secure, web‐based application designed to support data capture for research studies, providing: (1) an intuitive interface for validated data entry; (2) audit trails for tracking data manipulation and export procedures; (3) automated export procedures for seamless data downloads to common statistical packages; and (4) procedures for importing data from external sources.

Two researchers (R.M., G.E.) abstracted data on patient metrics, process measures, and patient outcomes from the VA Computerized Patient Record System (CPRS), VistA, and Joint Legacy Viewer (J.L.V.). To ensure data accuracy, 8% of charts were independently abstracted by both reviewers, and two randomly chosen variables (cancer recurrence, time to treatment) were assessed for inter‐reviewer agreement, yielding very‐good agreement (kappa 0.79 and 0.92).

### 2.6 Patient and health system characteristics

Patient characteristics at time of index surgery included age, sex, race (white, nonwhite), marital status, zip code, co‐morbidities (cardiovascular disease, obstructive pulmonary disease, type 2 diabetes, and chronic kidney disease), and >15 pounds of weight loss prior to the index surgery. Cardiovascular disease was defined as a history of systolic heart failure, coronary artery disease, myocardial infarction, stroke, transient ischemic event, peripheral artery disease, or peripheral vascular disease, or documentation of ejection fraction <50%. Obstructive pulmonary disease was defined as history of chronic obstructive pulmonary disease with or without supplemental oxygen use or a pulmonary function test with FEV1/FVC ratio of <0.80. Type 2 diabetes was defined as history of type 2 diabetes (regardless of any macro or microvascular complications) or an active hypoglycemic medication on the patient's medication list. Chronic kidney disease (CKD) was defined as history of CKD 3–5 in history or laboratory evidence of >3 months of glomerular filtration rate consistently <60 ml/min. From patient zip codes, median annual incomes were calculated using the American Community Survey Census Data 2007–2011^22^ and travel distances to the VHA site approximated using Google Maps.^23^ Health‐system covariates consisted of pre‐operative tumor board, assessment of ≥12 lymph nodes, index hospitalization length of stay (LOS), 30‐day re‐admission, and oncology referral, and were identified from physician notes, pathology reports, and consultation orders.

### 2.7 Statistical analyses

*STATA IC v.16.0* was used for all statistical analyses. Descriptive statistics were calculated on the baseline characteristics of patients by year‐group (2000–2005, 2006–2010, 2011–2015). The primary analysis considered the proportion of patients receiving AC (numerator) among all those eligible for AC by NCCN guidelines (denominator).

Multivariable logistic regression tested associations between year‐group and receipt of AC. Covariates were selected based on prior observed associations with receipt of AC or having suspected or known temporal changes during 2000–2015. Final multivariable model covariates were age, race, tumor stage, tumor type, travel distance, cardiovascular disease, length of stay, and 30‐day readmission. A subgroup analysis by cancer type (colon or rectal) was performed to evaluate for practice differences in treatment. As these subgroup models were over‐fitted for their sample size, they should be viewed as exploratory. Two post hoc exploratory analyses using chi square and student *t*‐tests evaluated whether patient and provider reasons for omitting AC changed over time. The first analysis considered all patients eligible for AC by NCCN guidelines. The second analysis restricted the denominator to only those patients who received an oncology evaluation and had a provider recommendation for AC.

## 3 RESULTS

### 3.1 Patient characteristics

**FIGURE 1:**
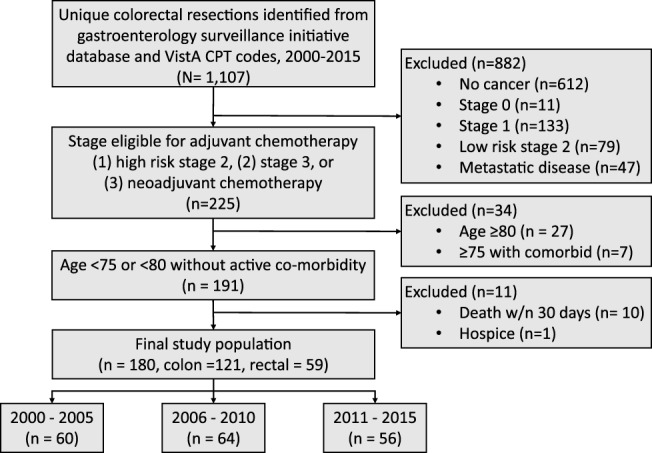
Flow of selected patient sample [Correction added on 24 August 2021, after first online publication: The figures 1 and 2 inadvertently swapped and have been corrected in this version.]

Of 1107 unique colorectal resections, 623 were excluded for no evidence of cancer, 212 for stage I or low‐risk stage II disease, 47 for metastatic disease, and 45 for age, active co‐morbidity, death, or hospice, yielding a final cohort of *N* = 121 colon and *N* = 59 rectal cancers (Figure 1). The number of patients in the analytic cohort and eligible for NCCN guideline concordant care who underwent colorectal surgery for high‐risk stage II or stage III CRC was similar across year‐groups; 2000–2005 (*N* = 60), 2006–2010 (*N* = 64), and 2011–2015 (*N* = 56).

Patients were predominantly male (96%) and white (79%) with a median age of 64 years (interquartile range [IQR], 59–68 years) (Table 1). Approximately half were married, with a median annual household income of $42.5 thousand (IQR $35.9–50.0 thousand) and similar proportions living <50 miles (36%), 55–99 miles (33%), and >100 miles (31%) from the VHA site. Approximately two‐thirds had at least one major co‐morbidity, including diabetes 34%, cardiovascular 32%, pulmonary 24%, and renal disease 4%.

**TABLE 1:** Patient characteristics

	Overall population *n* (%)	Year of surgery		
		2000–2005 *n* (%)	2006–2010 *n* (%)	2011–2015 *n* (%)
Total, *n*	180	60	64	56
**Patient demographics**				
Median age (IQR), years	64 (59–69)	64 (58–59)	63 (59–68)	65 (60–69)
Sex (male)	174 (96.7)	56 (96.6)	62 (96.9)	56 (96.6)
Race (white)	144 (80.0)	47 (81.0)	51 (79.7)	46 (79.3)
Married	91 (50.6)	28 (48.3)	33 (51.6)	30 (51.7)
Travel distance				
<50 miles	64 (35.6)	20 (34.5)	26 (40.6)	18 (31.0)
50–99 miles	60 (33.3)	20 (34.5)	15 (23.4)	25 (43.1)
≥100 miles	56 (31.1)	18 (31.0)	23 (35.9)	15 (25.8)
**Cancer characteristics**				
Colon	120 (66.7)	41 (70.7)	41 (64.1)	38 (65.5)
Rectal	60 (33.3)	17 (29.3)	23 (35.9)	20 (34.5)
Stage III	113 (62.8)	35 (60.3)	43 (67.2)	35 (60.3)
Stage II, high risk	66 (36.7)	23 (39.7)	21 (32.8)	22 (37.9)
**Patient health characteristics**				
Cardiovascular disease	64 (35.6)	22 (37.9)	20 (31.3)	22 (37.9)
Chronic kidney disease	10 (5.6)	6 (10.3)	2 (3.1)	2 (3.4)
Chronic obstructive pulmonary disease	44 (24.4)	19 (32.8)	10 (15.6)	15 (25.9)
Type 2 diabetes mellitus	61 (33.9)	18 (31.0)	21 (32.8)	22 (37.9)
Mean BMI (SD), kg/m^2^	28.2 (5.9)	27.3 (5.3)	27.9 (5.2)	29.4 (7.1)
Weight loss >15 lbs	41 (22.8)	18 (30.0)	11 (17.2)	12 (21.4)
**Health system characteristics**				
Preop tumor board	121 (67.2)	28 (48.3)	47 (73.4)	46 (79.3)
≥12 lymph nodes	124 (68.9)	30 (51.7)	44 (68.8)	50 (86.2)
Median LOS (IQR), days	7 (5–9)	7 (5–9)	7 (6–8.5)	7 (5–10)
Re‐admission, 30 day	27 (15.0)	7 (12.1)	10 (15.6)	10 (17.2)
Re‐operation, 30 day	12 (6.7)	1 (1.7)	5 (7.8)	6 (10.3)
Community Referral	13 (7.2)	0 (0)	12 (18.8)	1 (1.7)

Abbreviations: BMI, body mass index; IQR, interquartile range; LOS, Length of Stay; SD, standard deviation. Cardiovascular Disease, Systolic Heart Failure with Ejection Fraction <50, Coronary Artery Disease, Peripheral Artery Disease, Stroke or TIA; Chronic Kidney Disease, Glomerular Filtration Rate < 60 ml/min for more than 3 months; Chronic Obstructive Pulmonary Disease, Pulmonary Function Tests with FVC/FEV1 < 80; Type 2 Diabetes Mellitus, Documented in problem list or past medical history with or without complications.

### 3.2 Colorectal cancer care metrics

Median LOS for the index surgery hospitalization was 7 days (IQR 5–9 days) with no difference observed across year‐groups. Use of pre‐operative tumor board discussions improved over time; 48% between 2000 and 2005, 73% between 2006 and 2010, and 78% between 2011 and 2015. Improvements were also seen in the metric of ≥12 lymph nodes evaluated at time of surgery; 50% versus 69% versus 86% in the 3‐year groups.

### 3.3 Receipt of adjuvant chemotherapy

Of the 180 patients eligible to receive AC, 117 (65%) received chemotherapy (Table 2). Receipt of AC decreased over time; 72% in 2000–2005, 69% in 2006–2010, and 54% in 2011–2015. Compared to the 2000–2005 year‐group, patients undergoing surgery in 2011–2015 were significantly less likely to receive AC (odds ratio [OR] 0.35; 95% confidence interval [CI] 0.15–0.82). The exploratory subgroup analyses by cancer type showed similar results in both colon (OR 0.25 [0.08–0.74]) and rectal (OR 0.36 [0.47–2.7]) cancers for care received in 2011–2015 compared to 2000–2005.

**TABLE 2:** Receipt of adjuvant chemotherapy among eligible veterans with colorectal cancer, accounting for total, colon, and rectal cohorts. Counts of veterans receiving chemotherapy compared to eligible cohort assessed by primary predictor (year group) as well as cancer characteristics and operative factors. Multivariable analysis with adjusted odds ratio for receipt of adjuvant chemotherapy by primary predictor (year group), cancer characteristics, and operative factors

Receipt of adjuvant chemotherapy (Yes/No)						
Predictor	Number of patients receiving adjuvant chemotherapy/number eligible to receive adjuvant chemotherapy			Adjusted odds ratio^a^ (95% confidence interval)		
	Total	Colon	Rectal	Total	Colon	Rectal
**Year group**						
2000–2005	43/60	31/42	12/18	Reference	Reference	Reference
2006–2010	44/64	29/41	15/23	0.67 (0.29–1.6)	0.53 (0.17–1.6)	0.35 (0.05–2.5)
2011–2015	30/56	18/38	12/18	0.35 (0.15–0.82)	0.25 (0.08–0.74)	0.36 (0.47–2.7)
**Cancer characteristics**						
Stage II	34/66	11/31	23/35^b^	Reference	Reference	Reference
Stage III	83/114	67/90	16/24	3.0 (1.4–6.4)	6.2 (2.2–17.4)	0.90 (0.20–4.2)
Colon Cancer	78/121	78/121	0/0	Reference	N/A	N/A
Rectal Cancer	39/59	0/0	39/59	1.8 (0.78–4.1)	N/A	N/A
**Factors related to surgery**						
Length of Stay ≤7 days	75/110	58/87	17/23	Reference	Reference	Reference
Length of Stay >7 days	42/70	20/34	22/36	0.69 (0.33–1.4)	1.1 (0.40–2.8)	0.38 (0.08–1.7)
30‐day Readmission	13/25	9/16	4/9	0.46 (0.17–1.2)	0.68 (0.19–2.5)	0.17 (0.02–1.2)

^a^ Each of the three regression models adjusted for all variables in table along with age, race, and travel distance to VHA‐TVHS.

^b^ Includes *N* = 1 rectal cancer patient with stage 1 disease.

### 3.4 Decisions and workflows surrounding chemotherapy

**FIGURE 2:**
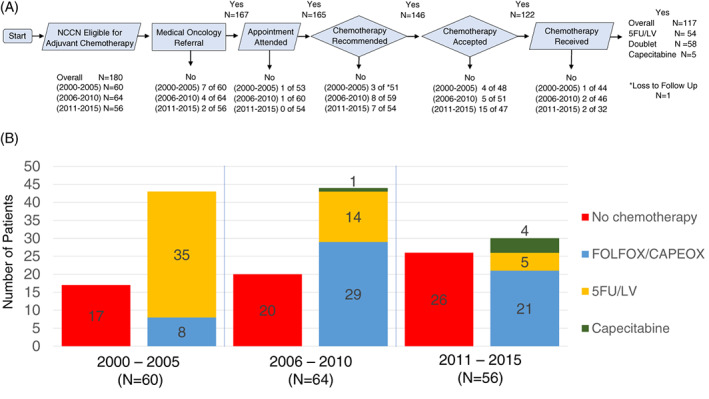
Chemotherapy workflows, decisions, and regimen selection. (A) Sequential post‐operative oncologic care and decisions resulting in omission or acceptance of AC. (B) Temporal trends in the selection of regimen choice. Definitions: 5FU, 5 fluorouracil; LV, leucovorin; CAPEOX, capecitabine + oxaliplatin; FOLFOX, 5‐flurouracil + leucovorin + oxaliplatin [Correction added on 24 August 2021, after first online publication: The figures 1 and 2 inadvertently swapped and have been corrected in this version.]

The most common reasons for not receiving AC were lack of medical oncology referrals (*N* = 13), oncology providers recommending against AC (*N* = 18), and patients declining AC (*N* = 24), with (*N* = 5) patients not receiving AC for unknown reasons, and (*N* = 1) having loss to follow up (Figure 2). There was an improvement in medical oncology referrals over time, with 7 of 60 (12%) in 2000–2005, 4 of 64 (6%) in 2006–2010, and 2 of 56 (4%) in 2011–2015 not having a referral (*p* < .001 for comparison of proportions). No patterns of change were observed in the proportion of providers not recommending AC.

Patient AC refusal increased over time. Evaluating for the full cohort eligible for AC (*N* = 180), 4 of 60 patients (6%) declined AC in 2000–2005, 5 of 60 (8%) declined in 2006–2010, and 15 of 56 (27%) declined during 2011–2015 (*p* = .002). Evaluating patient refusal using the denominator restricted to those patients who received an oncology visit and had a provider recommendation for AC (*N* = 146), demonstrated that 4 of 48 patients (8%) declined AC between 2000 and 2005, 5 of 51 (10%) declined between 2006 and 2010, and 15 of 47 (32%) declined AC between 2011 and 2015 (*p* < .001).

At the 5% significance level, no other factors (age, co‐morbidity, cancer stage, or type) were statistically associated with patients declining AC.

Regarding regimen choice, in the 2000–2005 year‐group, 5FU/LV was the predominant regimen (58%) followed by oxaliplatin doublets (13%) and capecitabine (0%). In 2006–2010, oxaliplatin doublets (45%), and capecitabine (1%) increased, while 5FU/LV decreased (23%). By 2011–2015, oxaliplatin doublets plateaued (37.5%) with minimal further increase in capecitabine (7%), but a continued decrease in 5FU/LV (9%) (Figure 2).

## 4 DISCUSSION AND CONCLUSIONS

This study identified a significant reduction in the receipt of AC among NCCN guideline‐eligible non‐elderly Veterans with CRC treated at our south regional VHA from 2011–2015 compared to 2000–2005. These results appeared to be due to more patients, regardless of age or cancer stage, declining AC, and occurred despite measurable improvements in pre‐operative tumor boards, quality lymph node evaluations at the time of surgery, and post‐operative medical oncology referrals. Consistent with prior randomized studies, lower rates of NCCN CRC guideline adherent care at the TVHS‐VHA during the 6‐month post‐operative period were associated with an increased rate of cancer recurrence and death.^24^

Our proportions of patients receiving AC between 2000–2005 (72%) and 2006–2010 (69%) are similar to those of the Surveillance, Epidemiology, and End Results (SEER) program estimates (72% and 66%)^1^ but are lower than the national VHA average of 77.5% observed from 2003–2006,^8^ and given our additional decrease over time, indicate an area for potential improvement.

Our findings add to several studies on AC quality for CRC. Using a SEER‐based US cohort, Murphy et al observed an initial increase in CRC AC utilization from 1995 to 2005 followed by a possible decrease in 2010 compared to 2005.^25^ Like Murphy et al, we identified an initial increase in oxaliplatin containing doublet therapies beginning in 2006 (corresponding with phase III studies observing improved OS versus 5FU/LV)^26^; however, this increase was surpassed by an even greater reduction in 5FU/LV. Then, in 2011–2015, when adjuvant CRC trials began focusing on ways to reduce oxaliplatin exposure to avoid long‐term neuropathic toxicities,^27^ we not only observed a slight decrease in FOLFOX and CAPEOX, but a continued decline in 5FU/LV. We hypothesize that providers may not be offering 5FU/LV as an effective alternative when a patient is not an optimal candidate for oxaliplatin. If this were true, it would be concerning given most of the survival benefit is due to 5FU/LV.

Walter et al observed decreased AC utilization among German patients treated in 2009–2012 compared to 2005–2008, driven primarily by patient refusal and age, especially with respect to oxaliplatin.^28^ Three additional studies documented patient refusal and age as major reasons for AC omission.^13^, ^28^, ^29^ Finally, Ko et al. demonstrated decreasing rates of AC acceptance from 2008 to 2010 among patients ≥75 years of age or with multiple co‐morbidities.^29^ Given the equivocal benefit in this study population (75+ with active co‐morbidity or 80+) our study specifically excluded this group yet still identified increasing refusal of AC among those without the above conditions.

Our study has limitations that should be noted. First, our study is retrospective and observational. As a cohort of predominantly white men, our VHA sample was underpowered to test for associations with sex and race and has limits on generalizability. The higher rates of co‐morbidities and rurality in our population likely biased our findings toward lower overall receipt of AC, however, these factors remained stable across the study period, and are unlikely to explain the temporal reduction in AC.

It is possible that unmeasured confounders impacted our findings. For example, our study did not account for co‐insurance. Prior to the 2008 recession, approximately 77% of Veterans were co‐insured with non‐VHA insurance, however, the number of Veterans enrolling or utilizing VA services increased during 2009–2013 (particularly in non‐Medicaid expanding states).^30^ While our study attempted to address numerous local VHA practice changes directly pertinent to CRC AC such as pre‐operative tumor boards and intra‐operative lymph node assessments. It is possible that other unmeasured practice level changes such as oncology personnel, visit time, or quality of risk/benefit discussions contributed to greater AC refusal among patients. Such practice changes might account for provider recommendations against adjuvant chemotherapy. Most provider omissions cited advanced patient age, co‐morbidities, and poor post‐operative recovery and these reasons remained stable over the 2000–2015 time‐period. Data on visit length, the number and type of visits with each provider or content of individual risk/benefit conversations could not be obtained through retrospective chart abstraction.

It is possible that 5FU and LV drug shortages contributed to the reduction in AC. 5FU and LV are two of the more common chemotherapies with shortages, and oncology providers often respond to drug shortages by substituting, delaying, or even omitting therapy.^31^ National VHA drug shortages may have contributed to our findings in the 2011–2015 year‐group, however, patients who declined AC between 2011 and 2015 were not specific to any periods of drug shortages.

Our findings raise the possibility that declining rates of CRC AC may not simply be a phenomenon of frail and elderly patients, but a more general trend occurring across ages driven primarily by factors influencing patient attitudes toward AC. While these specific factors remain unknown, they do not appear to be related to worsening Veteran health status nor do they impact other CRC post‐operative care such as surgical follow up or colonoscopies.^24^ Examples of such patient factors might include diminished trust in medical professionals and experts, increased concerns over costs and toxicities of chemotherapy, greater reliance on alternative therapies or sources of information, and an inadequate understanding of risks and benefits. Similarly, changes in patient/provider shared decision‐making conversations regarding the risks and benefits of AC may be impacting acceptance. Factors influencing these conversations could involve clinic time, personnel, setting, and regimen selection. Specifically, physician conveyance of concerns related to oxaliplatin toxicity may change the acceptability and logistics of chemotherapy for patients. With recent studies observing noninferiority for 3 versus 6 months CAPEOX in subsets of patients with HR Stage II/IIIA disease,^32^ assessing duration and completion of chemotherapy will be important for future observational studies.

In summary, our study provides several novel insights on how quality care patterns can change over time and how reliance on national‐level data can lead to underestimation of important local barriers and facilitators that are critical to improving high quality cancer care. Future work should investigate underlying reasons why patients chose not to receive AC despite meeting NCCN eligibility and whether our observed reduction in AC is specific to our institution and/or CRC or reflects a broader trend of patients declining potentially curative chemotherapy.

## AUTHOR CONTRIBUTIONS

**Gretchen Edwards:** Conceptualization; data curation; formal analysis; investigation; methodology; writing‐review & editing. **Lauren Samuels:** Conceptualization; formal analysis; methodology; software; visualization; writing‐review & editing. **Cathy Eng:** Formal analysis; supervision; validation; writing‐review & editing. **Christianne Roumie:** Conceptualization; methodology; project administration; resources; supervision; writing‐review & editing.

## CONFLICT OF INTEREST

The authors have no conflicts of interest to report.

## ETHICS STATEMENT

The VHA Institutional Review Board (IRB) and Research and Development Committee approved this study, with a waiver for subject consent under secondary data review.

## Data Availability

The data that support the findings of this study are available on request from the corresponding author. The data are not publicly available due to privacy or ethical restrictions.
